# Development of a structural growth curve model that considers the causal effect of initial phenotypes

**DOI:** 10.1186/s12711-019-0461-y

**Published:** 2019-05-02

**Authors:** Akio Onogi, Atsushi Ogino, Ayako Sato, Kazuhito Kurogi, Takanori Yasumori, Kenji Togashi

**Affiliations:** 1 Institute of Crop Science, National Agriculture and Food Research Organization, 2-1-2 Kannondai, Tsukuba, Ibaraki 305-8518 Japan; 2 Maebashi Institute of Animal Science, Livestock Improvement Association of Japan, Inc, Maebashi, 371-0121 Japan; 3 Cattle Breeding Department, Livestock Improvement Association of Japan, Inc, Tokyo, 135-0041 Japan

## Abstract

**Background:**

Growth curves have been widely used in genetic analyses to gain insights into the growth characteristics of both animals and plants. However, several questions remain unanswered, including how the initial phenotypes affect growth and what is the duration of any such impact. For beef cattle production in Japan, calves are procured from farms that specialize in reproduction and then moved to other farms where they are fattened to achieve their market/purchase value. However, the causal effect of growth, while calves are on the reproductive farms, on their growth during fattening remains unclear. To investigate this, we developed a model that combines a structural equation with a growth curve model. The causal effect was modeled with B-splines, which allows inference of the effect as a curve. We fitted the proposed structural growth curve model to repeated measures of body weight from a Japanese beef cattle population (n = 3831) to estimate the curve of the causal effect of the calves’ initial weight on their trajectory of growth when they are on fattening farms.

**Results:**

Maternal and reproduction farm effects explained 26% of the phenotypic variance of initial weight at fattening farms. The structural growth curve model was fitted to remove the effects of these factors in growth curve analysis at fattening farms. The estimated curve of causal effects remained at approximately 0.8 for 200 d after the calves entered the fattening farms, which means that 64% of the phenotypic variance was explained by the initial weight. Then, the effect decreased linearly and disappeared approximately 620 d after entering the fattening farms, which corresponded to an average age of 871.5 d.

**Conclusions:**

The proposed model is expected to provide more accurate estimates of genetic values for growth patterns because the confounding causal factors such as maternal and reproduction farm effects are removed. Moreover, examination of the inferred curve of the causal effect enabled us to estimate the effect of a calf’s initial weight at arbitrary times during growth, which could provide suitable information for decision-making when shifting the time of slaughter, building models for genetic evaluation, and selecting calves for market.

**Electronic supplementary material:**

The online version of this article (10.1186/s12711-019-0461-y) contains supplementary material, which is available to authorized users.

## Background

Fitting curves to longitudinal phenotypic data is a common methodology that is used in animal and plant genetics to gain insights into individual growth patterns. Research on growth curves has a long history, and to date a number of curves have been used to model growth, including logistic [1], Richards [2], Gompertz [3], von Bertalanffy [4], and Brody [5] curves. These curves include three to four parameters that are often regarded as new traits and are subjected to various genetic analyses, such as the estimation of genetic parameters or mapping of quantitative trait loci [6–10], to better understand the genetic architecture of growth patterns.

Regarding growth curve analysis, it is important to understand the impact of initial measures on subsequent growth and the duration of this impact. For example, birth weight is affected by various factors such as maternal effects and the environment, which can have causal effects on subsequent growth. For beef cattle production in Japan, calves are usually born on farms that specialize in reproduction, and then at about 9 months of age they are moved to other farms for fattening to achieve their market value at around 30 months of age. However, although the phenotypes of the calves when they enter the fattening farms are known to influence their growth patterns during the fattening phase, the duration of this impact is unclear. The causal effect of a phenotype such as initial weight will interfere with growth curve analyses if it is affected by factors that are not considered in the growth curves, such as maternal effects.

Therefore, to address this issue, we developed a growth curve model that considers the causal effect of initial weight by combining a structural equation for causality inference with growth curves in a Bayesian framework. In quantitative genetics, structural equations are often used to infer causal relationships between phenotypes in multivariate mixed models [11–13]. In the current study, we applied structural equation modeling to longitudinal data and inferred the causal effect of the initial phenotypic value as a curve over time by using B-splines. We fitted this structural growth curve (SGC) model to real data on weight from a beef cattle population in Japan to reveal the causal effect of calf weight at entry to the fattening farms.

## Methods

### Data

**Fig. 1:**
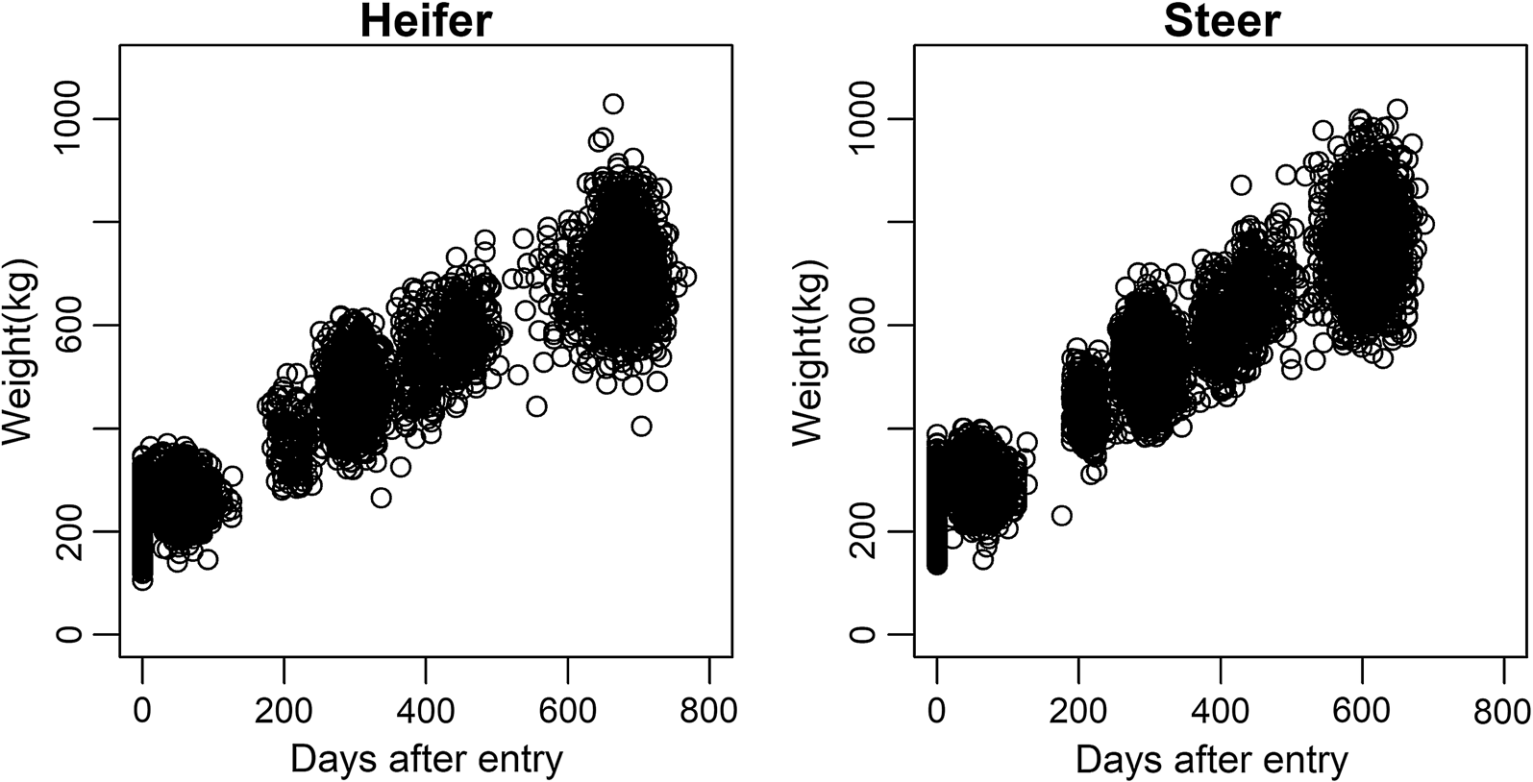
Distribution of weights in Japanese black heifers and steers. The day of entry to the experimental station (the day of the first measurement) was set as the initial day (day 0)

The Livestock Improvement Association of Japan, Inc. (LIAJ) measured the weight of 3831 Japanese black cattle, a major beef cattle breed in Japan, as part of their progeny-testing program (see Table 1 for a data summary). These animals comprised 1600 heifers and 2231 steers born between 2006 and 2013 on 1845 farms, which were moved to three experimental stations for fattening at a mean (± SD) age of 251.5 (± 20.4) d. After fattening, these animals were slaughtered at a mean age of 886.8 ± 46.7 d. The weight of each animal was measured on the day of entry to the station and several times during fattening and before shipment for slaughter (weight at slaughter). One to six weight records were available per animal during the fattening period (mean = 4.4 ± 0.7). It should be noted that the frequency and age of measurement differed between stations and years, which resulted in a dispersed distribution of weight records after entry to the stations (Fig. 1). All animals were reared and slaughtered according to the Japanese rules and regulations for animal care.

**Table 1:** Data summary

Characteristic	Value^a^
Number of animals with weight records	3831 (1600 heifers and 2231 steers)
Number of animals in the numerator relationship matrix	24,284
Mean number of weight records per animal	4.4 ± 0.7
Number of reproduction farms	1845
Number of experimental stations (fattening farms)	3
Average age at entry to the stations (d)	251.5 ± 20.4
Average age at slaughter (d)	886.8 ± 46.7
Average weight on the day of entry to the stations (kg)	236.0 ± 39.0 (heifers: 221.3 ± 35.4; steers: 246.6 ± 38.0)
Average weight at slaughter (kg)	729.8 ± 81.5 (heifers: 699.2 ± 76.5; steers: 751.8 ± 77.7)

Animals were born on reproduction farms and moved to the experimental stations (fattening farms) at an average age of 251.5 d. The weight of each animal was first measured on the day of entry to the stations. Because the frequency and age of measurement differed between stations and years, weights had a dispersed distribution across the days after entry, as shown in Fig. 1

^a^Mean values are presented ± SD

### Structural growth curve model

In the SGC model, the phenotypes of the trait of interest are chronologically measured at time $t = 0, 1, \ldots , T$, and the phenotypic values at $t = 0$ have a causal effect on the phenotypes at subsequent times. The SGC model for individual $i$ can be represented by the following two-equation system:

1 $$Y_{i,0} = {\mathbf{x}}_{{{\mathbf{i}},{\mathbf0}}} {\varvec{\upbeta}}_{\mathbf0} + u_{i,0} + \mathop \sum \limits_{j = 1}^{J} v_{j,i} + e_{i,0} ,$$

2 $$Y_{i,t} = Y_{i,0} \lambda \left( t \right) + f\left( {t,A_{i} ,B_{i} ,K_{i} } \right) + e_{i,t} ,$$

where $Y_{i,0}$ and $Y_{i,t}$ are the phenotypic values at times $0$ and $t$, respectively; ${\mathbf{x}}_{{{\mathbf{i}},{\mathbf0}}}$ is the incidence row vector; ${\varvec{\upbeta}}_{\mathbf0}$ is the vector of fixed effects; $u_{i,0}$ is the additive genetic effect; $v_{j,i}$ is a random effect, such as a maternal effect; $J$ is the number of random effects other than the additive genetic effect; $e_{i,0}$ and $e_{i,t}$ are the residuals; $\lambda \left( t \right)$ is the magnitude of the causal effect of $Y_{i,0}$ at time $t$; and $f$ is the function of the growth curve with parameters $A_{i}$, $B_{i}$ and $K_{i}$. In this study, ${\mathbf{x}}_{{{\mathbf{i}},{\mathbf0}}}$ included sex, birth year, and birth season (winter, spring, summer, and fall), as well as age, which was standardized and added as a covariate. The maternal effect and the effects of the reproductive farms were added as $v_{1}$ and $v_{2}$, respectively. The Gompertz function was used for $f$:

3 $$f\left( {t,A_{i} ,B_{i} ,K_{i} } \right) = A_{i} { \exp }\left\{ { - B_{i} { \exp }\left( { - K_{i} t} \right)} \right\},$$

where $A_{i}$ represents the asymptotic value at $t = \infty$, $B_{i}$ is a scaling parameter that shifts the curves back and forth, and $K_{i}$ represents the maximum growth speed (i.e., maturing rate).

These growth curve parameters have a hierarchical structure:

4 $$A_{i} = {\mathbf{x}}_{{\mathbf{i}}} {\varvec{\upbeta}}_{{\mathbf{A}}} + u_{i,A} + e_{i,A} ,$$

5 $$B_{i} = {\mathbf{x}}_{{\mathbf{i}}} {\varvec{\upbeta}}_{{\mathbf{B}}} + u_{i,B} + e_{i,B} ,$$

6 $$K_{i} = {\mathbf{x}}_{{\mathbf{i}}} {\varvec{\upbeta}}_{{\mathbf{K}}} + u_{i,K} + e_{i,K} ,$$

where ${\mathbf{x}}_{{\mathbf{i}}}$ represents the incidence row vector; ${\varvec{\upbeta}}_{{\mathbf{A}}}$, ${\varvec{\upbeta}}_{{\mathbf{B}}}$, and ${\varvec{\upbeta}}_{{\mathbf{K}}}$ are the vectors of fixed effects; $u_{i,A}$, $u_{i,B}$, and $u_{i,K}$ are the additive genetic effects; and $e_{i,A}$, $e_{i,B}$, and $e_{i,K}$ are the random residual effects. In this study, ${\mathbf{x}}_{{\mathbf{i}}}$ included sex and station. The additive genetic effects ${\mathbf{u}}_{{\mathbf{A}}} = \left[ {u_{i,A} } \right]$, ${\mathbf{u}}_{{\mathbf{B}}} = \left[ {u_{i,B} } \right]$, ${\mathbf{u}}_{{\mathbf{K}}} = \left[ {u_{i,K} } \right]$, and ${\mathbf{u}}_{\mathbf0} = \left[ {u_{i,0} } \right]$ for $i = 1, \ldots , N$, where $N$ is the number of animals, follow a multivariate normal distribution:

7 $$\left[ {\begin{array}{*{20}c} {\begin{array}{*{20}c} {{\mathbf{u}}_{{\mathbf{A}}} } \\ {{\mathbf{u}}_{{\mathbf{B}}} } \\ \end{array} } \\ {{\mathbf{u}}_{{\mathbf{K}}} } \\ {{\mathbf{u}}_{\mathbf0} } \\ \end{array} } \right]\sim{\text{N}}\left( {\mathbf{0},{\varvec{\Sigma}}_{{\mathbf{u}}} \otimes {\mathbf{A}}_{{\mathbf{u}}} } \right),$$

where ${\varvec{\Sigma}}_{{\mathbf{u}}}$ is the 4 × 4 genetic covariance, $\otimes$ represents the Kronecker product, and ${\mathbf{A}}_{{\mathbf{u}}}$ is the additive genetic relationship matrix. The random effect ${\mathbf{v}}_{{\mathbf{j}}} = \left[ {\nu_{j,i} } \right]$ for $i = 1, \ldots , N$ also follows a multivariate normal distribution:

8 $${\mathbf{v}}_{{\mathbf{j}}} \sim {\text{N}}\left( {\mathbf{0},{\mathbf{H}}_{{{\mathbf{vj}}}}\upsigma_{\text{vj}}^{2} } \right),$$

where $\upsigma_{\text{vj}}^{2}$ is the variance component and ${\mathbf{H}}_{{{\mathbf{vj}}}}$ is the matrix defining the covariance structure among individuals, which can be the additive genetic relationship or the identity matrix. In this study, we used a numerator relationship matrix for ${\mathbf{A}}_{{\mathbf{u}}}$ and ${\mathbf{H}}_{{{\mathbf{v}}1}}$ (i.e., relationship matrices for the animal and maternal effects) that was generated by using pedigree records that went back five generations (involving 24,284 animals) and an identity matrix for ${\mathbf{H}}_{{{\mathbf{v}}2}}$ (matrix for the reproductive farm effect).

The residuals were assumed to follow a multivariate normal distribution or normal distributions:

9 $$\left[ {\begin{array}{*{20}c} {{\mathbf{e}}_{{\mathbf{A}}} } \\ {{\mathbf{e}}_{{\mathbf{B}}} } \\ {{\mathbf{e}}_{{\mathbf{K}}} } \\ \end{array} } \right]\sim {\text{N}}\left( {\mathbf{0},{\varvec{\Sigma}}_{{\mathbf{e}}} \otimes {\mathbf{I}}} \right),$$

10 $${\text{e}}_{{{\text{i}},0}} \sim {\text{N}}\left( {0,\upsigma_{\text{eo}}^{2} } \right),$$

11 $${\text{e}}_{{{\text{i}},{\text{t}}}} \sim {\text{N}}\left( {0,\upsigma_{\text{e}}^{2} } \right).$$

The function $\lambda \left( t \right)$ was modeled using a B-spline, as developed in a previous study [14], and can be written as:

12 $$\lambda \left( t \right) = \mathop \sum \limits_{j = 0}^{{N_{p} - 1}} P_{j} S_{j} \left( t \right),$$

where $N_{p}$ is the number of splines, $P_{j}$ is the weight of the $j$-th spline, and $S_{j} \left( t \right)$ represents the cubic spline. We determined the number of splines and the positions of knots based on previously used methods [14]. Briefly, we set $N_{p}$ to 8 (indicating 12 knots) and set the first and last four knots at $t = 0$ and $5T_{L} /4$, respectively, where *T*_*L*_ is the time point of the last measurement. These knots were repeated to constrain the span of B-splines [15]. The remaining knots were set at $t = T_{L} /4,$ $T_{L} /2,3T_{L} /4$, and $T_{L}$, respectively.

The likelihood of the SGC model was derived based on a method that was previously used in structural equation modeling [16]. Equations (1) and (2) can be written as:

13 $$Y_{i,0} = {\mathbf{x}}_{{{\mathbf{i}},{\mathbf0}}} {\varvec{\upbeta}}_{\mathbf0} + u_{i,0} + \mathop \sum \limits_{j = 1}^{J} v_{j,i} + e_{i,0} ,$$

14 $$Y_{i,t} - Y_{i,0} \lambda \left( t \right) = f\left( {t,A_{i} ,B_{i} ,K_{i} } \right) + e_{i,t} .$$

Then, the left-hand side of each of these equations can be combined in the matrix form:

15 $$\left[ {\begin{array}{*{20}c} {\begin{array}{*{20}c} {\begin{array}{*{20}c} 1 &\quad 0 & \quad \ldots \\ { - \lambda \left( 1 \right)} &\quad 1 &\quad \ddots \\ \vdots &\quad \ddots &\quad 1 \\ \end{array} } \\ {\begin{array}{*{20}c} { - \lambda \left( T \right)} & \quad \ldots &\quad 0 \\ \end{array} } \\ \end{array} } & {\begin{array}{*{20}c} {\begin{array}{*{20}c} 0 \\ \vdots \\ \end{array} } \\ 0 \\ 1 \\ \end{array} } \\ \end{array} } \right]\left[ {\begin{array}{*{20}c} {\begin{array}{*{20}c} {Y_{i,0} } \\ {Y_{i,1} } \\ \vdots \\ \end{array} } \\ {Y_{i,T} } \\ \end{array} } \right] = {\varvec{\Lambda}}{\mathbf{Y}}_{{\mathbf{i}}}.$$

Because the residuals $e_{i,0}$ and $e_{i,t}$ are assumed to be independent (Eqs. 10 and 11), the density of ${\varvec{\Lambda}} {\mathbf{Y}}_{{\mathbf{i}}}$ for $i = 1, \ldots , N$ can be expressed as:

16 $$\mathop \prod \limits_{i = 1}^{N} \left( {\upsigma_{{{\text{e}}0}}^{2} } \right)^{{ - \frac{1}{2}}} { \exp }\left[ { - \frac{1}{{2\upsigma_{{{\text{e}}0}}^{2} }}\left( {Y_{i,0} - {\mathbf{x}}_{{{\mathbf{i}},{\mathbf0}}} {\varvec{\upbeta}}_{\mathbf0} - u_{i,0} - v_{i} } \right)^{2} } \right] \times \mathop \prod \limits_{i = 1}^{N} \mathop \prod \limits_{t = 1}^{T} \left( {\upsigma_{\text{e}}^{2} } \right)^{{ - \frac{1}{2}}} { \exp }\left\{ { - \frac{1}{{2\upsigma_{\text{e}}^{2} }}\left[ {Y_{i,t} - \lambda \left( t \right)Y_{i,0} - {\text{f}}\left( {t,A_{i} ,B_{i} ,K_{i} } \right)} \right]^{2} } \right\}.$$

Then, the density of ${\mathbf{Y}}_{{\mathbf{i}}}$ can be obtained by transforming ${\varvec{\varLambda}} {\mathbf{Y}}_{{\mathbf{i}}}$ to ${\mathbf{Y}}_{{\mathbf{i}}}$. Because the determinant of the Jacobian of the transformation (i.e., $\left| {\varvec{\Lambda}} \right|$) is 1, the likelihood of the SGC model is also given by Eq. (16). Note that because the time points of the measurements of the animals included in our study can vary between animals, $t$ and $T$ were indexed by $i$ (i.e., $t_{i}$ and $T_{i}$, respectively). Also note that $T_{L}$ is the greatest value among all $T_{i}$.

The prior distributions of the fixed effects were assumed to be proportional to constant values. For $\upsigma_{\text{vj}}^{2}$, $\upsigma_{{{\text{e}}0}}^{2}$, and $\upsigma_{\text{e}}^{2}$, a non-informative scaled inverse Chi squared distribution was applied: $\chi^{ - 2} \left( { - 2,0} \right)$. For ${\varvec{\Sigma}}_{{\mathbf{u}}}$ and ${\varvec{\Sigma}}_{{\mathbf{e}}}$, inverse Wishart distributions were applied: ${\varvec{\Sigma}}_{{\mathbf{u}}} \sim{\text{IW}}\left( {\nu_{u} ,{\mathbf{S}}_{{\mathbf{u}}} } \right)$ and ${\varvec{\Sigma}}_{{\mathbf{e}}} \sim{\text{IW}}\left( {\nu_{e} ,{\mathbf{S}}_{{\mathbf{e}}} } \right)$, respectively, where $\nu_{u}$ and $\nu_{e}$ were set to 6, and ${\mathbf{S}}_{{\mathbf{u}}}$ and ${\mathbf{S}}_{{\mathbf{e}}}$ were determined as described in the next section (Comparison of models). For the weights of B-splines, $P_{j}$, we assumed normal distributions:

17 $$P_{0} \sim {\text{N}}\left( {0,1000\upsigma_{\text{p}}^{2} } \right),$$

18 $$P_{1} \sim {\text{N}}\left( {0,1000\upsigma_{\text{p}}^{2} } \right),$$

19 $$P_{j} \sim {\text{N}}\left( {2P_{j - 1} - P_{j - 2} ,\upsigma_{\text{p}}^{2} } \right)\quad {\text{for}}\,\, j \ge \, 2.$$

The variances of $P_{0}$ and $P_{1}$ were multiplied by an arbitrary value of 1000 to make the prior distributions vague, and $\chi^{ - 2} \left( { - 2,0} \right)$ was applied to the prior distribution of $\upsigma_{\text{p}}^{2}$.

### Comparison of models

We compared the SGC model with the ordinal growth curve model, which does not consider causal effects. This model can be described by:

20 $$Y_{i,t} = f\left( {t,A_{i} ,B_{i} ,K_{i} } \right) + e_{i,t} .$$

The hierarchical structure of this model is the same as that of the SGC model except that:

21 $$\left[ {\begin{array}{*{20}c} {{\mathbf{u}}_{{\mathbf{A}}} } \\ {{\mathbf{u}}_{{\mathbf{B}}} } \\ {{\mathbf{u}}_{{\mathbf{K}}} } \\ \end{array} } \right]\sim {\text{N}}\left( {\mathbf{0},{\varvec{\Sigma}}_{{\mathbf{u}}}^{\varvec{*}} \otimes {\mathbf{A}}_{{\mathbf{u}}} } \right),$$

where ${\varvec{\Sigma}}_{{\mathbf{u}}}^{\varvec{*}}$ is the 3 × 3 genetic covariance. We assumed that ${\varvec{\Sigma}}_{{\mathbf{u}}}^{\varvec{*}} \sim{\text{IW}}\left( {v_{u}^{*} ,{\mathbf{S}}_{{\mathbf{u}}}^{*} } \right)$, where $v_{u}^{*}$ was equal to 5, and ${\mathbf{S}}_{{\mathbf{u}}}^{*}$ was determined as outlined below. This model is similar to those used in previous studies [17–19].

When fitting the SGC model to the data, the day of entry to the experimental stations was set as the initial day ($t = 0$). When fitting the ordinal growth curve model to the data, either the day of entry or the day of birth was set as the initial day [referred to as the growth curve model fitted to the entry day data (GC_A) and birth day data (GC_B), respectively]. The unit of time was d in each model. We compared these models using the mean log-likelihood, the deviance information criterion (DIC) [20], and the widely applicable information criterion (WAIC) [21].

First, we fitted the GC_B model to the data using the scaling parameters ${\mathbf{S}}_{{\mathbf{u}}}^{\varvec{*}}$ and ${\mathbf{S}}_{{\mathbf{e}}}$, which were arbitrarily determined as:

22 $${\mathbf{S}}_{{\mathbf{u}}}^{\varvec{*}} = {\mathbf{S}}_{{\mathbf{e}}} = \left[ {\begin{array}{*{20}c} {1{\text{e}} + 4} & 0 & 0 \\ 0 & {0.65} & 0 \\ 0 & 0 & {5{\text{e}} - 7} \\ \end{array} } \right].$$

In the final analyses, ${\mathbf{S}}_{{\mathbf{u}}}^{\varvec{*}}$, and ${\mathbf{S}}_{{\mathbf{e}}}$ were set following the posterior means of the preliminary analysis. In addition, we fitted a linear mixed-effect model, which was the same as Eq. 1 in the SGC model, to the entry day weight using airemlf90 ver. 1.103 [22], with the default value of 1e−10 as the convergence criterion. ${\mathbf{S}}_{{\mathbf{u}}}$ was determined from the estimates provided by these two preliminary analyses (the GC_B and linear mixed models for the weight on entry day). The off-diagonal elements in ${\mathbf{S}}_{{\mathbf{u}}}$ that corresponded to the covariances between ${\mathbf{u}}_{\mathbf0}$ and ${\mathbf{u}}_{{\mathbf{A}}}$, ${\mathbf{u}}_{{\mathbf{B}}}$, and ${\mathbf{u}}_{{\mathbf{K}}}$ were determined as the empirical covariances of these random effects obtained from the two preliminary analyses.

### Estimation of parameters

The parameters in the SGC, GC_A, and GC_B models were estimated using the Markov chain Monte Carlo (MCMC) method. Gibbs sampling could be applied to all of the parameters except $A_{i}$, $B_{i}$, $K_{i}$, and $P_{j}$. ${\varvec{\upbeta}}_{\mathbf0}$, ${\varvec{\upbeta}}_{{\mathbf{A}}}$, ${\varvec{\upbeta}}_{{\mathbf{B}}}$, and ${\varvec{\upbeta}}_{{\mathbf{K}}}$ had normal posterior distributions, whereas ${\mathbf{v}}_{{\mathbf{j}}}$, and ${\mathbf{u}}_{{\mathbf{A}}}$, ${\mathbf{u}}_{{\mathbf{B}}}$, ${\mathbf{u}}_{{\mathbf{K}}}$, and ${\mathbf{u}}_{0}$ had multivariate normal posterior distributions. The posterior distributions of ${\varvec{\upbeta}}_{{\mathbf{A}}}$, ${\varvec{\upbeta}}_{{\mathbf{B}}}$, ${\varvec{\upbeta}}_{{\mathbf{K}}}$, ${\mathbf{u}}_{{\mathbf{A}}}$, ${\mathbf{u}}_{{\mathbf{B}}}$, and ${\mathbf{u}}_{{\mathbf{K}}}$ could be derived by considering the growth curve parameters ($A_{i}$, $B_{i}$ and $K_{i}$) as response variables. $\upsigma_{\text{vj}}^{2}$, $\upsigma_{{{\text{e}}0}}^{2}$, $\upsigma_{\text{e}}^{2}$, and $\upsigma_{\text{p}}^{2}$ had scaled inverse Chi squared posterior distributions, while ${\varvec{\Sigma}}_{{\mathbf{u}}}$, ${\varvec{\Sigma}}_{{\mathbf{u}}}^{\varvec{*}}$, and ${\varvec{\Sigma}}_{{\mathbf{e}}}$ had inverse Wishart posterior distributions, all of which were derived following a previous study [23]. Since the posterior distributions of $A_{i}$, $B_{i}$, $K_{i}$, and $P_{j}$ were not closed form expressions, Metropolis–Hastings sampling was applied by adopting a random-walk algorithm.

The number of iterations for the SGC model was 2.5 million with the first 2 million being discarded. By contrast, there were 1 million iterations for both the GC_A and GC_B models, with the first 0.6 million being discarded. The sampling interval was 10 for each model. We ran three chains with different initial values and checked the convergence of MCMC, as described previously [24].

### Parametric bootstrapping

To evaluate the accuracy of the parameter estimation of the SGC model, parametric bootstrapping was conducted. The weight at slaughter of each animal was simulated using the estimates of the variance components, the fixed effects, and the causal effect generated by the SGC model. A single-trait animal model that included sex and experimental station as fixed effects and age as a covariate was then fitted to the simulated weights to estimate the heritability at slaughter. This procedure was repeated 1000 times and the heritability that was estimated from the simulated data was compared with that estimated from the real data.

## Results and discussion

### Convergence of the MCMC chains

The $\hat{R}$ statistics of the convergence diagnosis [24] were calculated for the log-likelihood values and the parameters for each model. The $\hat{R}$ statistics decreased to 1.0 as the MCMC chains converged. The statistics for the log-likelihood values were 1.047, 1.060, and 1.029 for the SGC, GC_A, and GC_B models, respectively; these values were lower than 1.1, which was previously suggested to be a rough threshold [24]. Most $\hat{R}$ statistics for the parameters were also lower than the threshold, with the exception of a few parameters in the SGC model, including $P_{5}$ (1.153), $P_{6}$ (1.185), and $P_{7}$ (1.148), which are the weights of splines, and the additive genetic variances for parameters $A$ (1.122) and $Y_{i,0}$ (1.127). However, the statistics for these parameters are close to 1.1, indicating that the MCMC chains for each model converged to stationary distributions. The SGC model took more than three times as many iterations as the other models to reach convergence (2 million vs. 0.6 million), which may be due to the model complexity.

### Comparison of models using information criteria

The mean log-likelihood and two information criteria (DIC and WAIC) were calculated for each model (Table 2). The mean log-likelihood was much higher and the information criteria were much lower for the SGC and GC_A models, in which the entry day was set as the initial day, than for the GC_B model, in which the day of birth was set as the initial day. Thus, it appears that setting the entry day as the initial day provides a better description of the patterns of weight change. This result was expected because the GC_B model covered the growth periods on both the reproduction and fattening farms without considering the transition between the two, while the SGC and GC_A models circumvented this issue by setting the entry day as the initial day. In addition, the mean log-likelihood was higher and the information criteria were lower for the SGC model than for the GC_A model (Table 2), which suggests that the initial weight had a causal effect on subsequent growth that was effectively considered in the SGC model.

**Table 2:** Mean log-likelihood and information criteria for each model

Model	Mean log-likelihood	DIC^d^	WAIC^e^
SGC^a^	− 5,523,358.6	7,871,834.7	12,466,275.7
GC_A^b^	− 5,574,092.6	7,872,045.8	12,567,909.0
GC_B^c^	− 7,335,939.9	10,341,550.1	16,537,364.6

^a^Structural growth curve model fitted to the data with the day of entry to the experimental station set as the initial day

^b^Growth curve model fitted to the data with the day of entry to the experimental station set as the initial day

^c^Growth curve model fitted to the data with the day of birth set as the initial day

^d^Deviation information criterion

^e^Widely applicable information criterion

### Causal effect of the initial weight

**Fig. 2:**
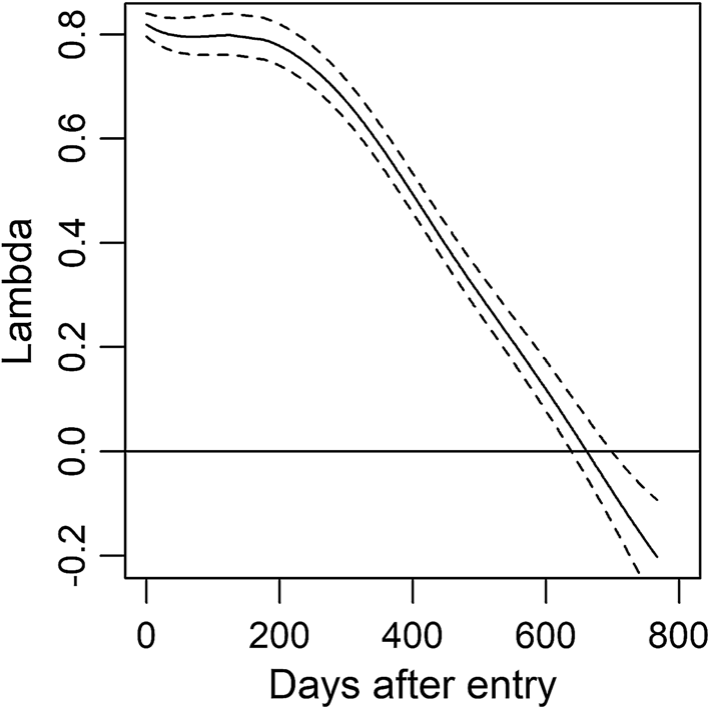
Estimated causal effect of the initial weight on the weight of Japanese black cattle during the fattening period. The *y*-axis denotes the magnitude of the causal effect. The day of entry to the experimental station was set as the initial day (day 0). Solid line: posterior mean; dotted line: 95% highest posterior density interval

The curve of the causal effect of the initial weight, $\lambda \left( t \right)$, that was inferred by the SGC model is shown in Fig. 2. $\lambda \left( t \right)$ remained at approximately 0.8 for 200 d after the calves had entered in the stations, then it exhibited a linear decrease until it disappeared at about day 620 after entry. Although many animals (1685 of the 3831 animals) were slaughtered before this time point, the causal effect on slaughter age was only 0.064 ± 0.058, which suggests that approximately 0.4% of the phenotypic variance of slaughter weight was explained by the variance of the initial weight (derived as the square of the average magnitude).

The causal effect of initial measures estimated as $\lambda \left( t \right)$ can provide valuable information when building models for genetic evaluation in progeny-testing programs: if the causal effect of the initial weight is high, the reproduction farm and maternal effects will be significant at the time of slaughter, because these effects represented a certain proportion of the variances of the initial weight (Table 3). Consequently, the reproduction farm and maternal effects need to be included in the evaluation model. The evaluation model for carcass traits of the Japanese black cattle breed that is currently used by LIAJ includes the effects of sex, fattening farm, birth month and year, and age as fixed effects but does not consider reproduction farm and maternal effects. Our results justify the use of such a model for the evaluation of carcass traits in this breed because the causal effect of the initial weight is not significant. Although this can be assessed by fitting ordinal mixed models to data obtained during the period of interest (e.g., between 200 and 300 d after entry), a key strength of the SGC model is that the length of the period that may affect the inference does not need to be determined.

**Table 3:** Variance components of the initial weight estimated by the structural growth curve model

	Additive genetic effect	Maternal effect	Reproduction farm effect	Residual
Variance	499.4 (432.3, 568.7)	49.6 (10.3, 90.5)	219.0 (180.8, 259.1)	265.0 (218.0, 310.7)
Proportion to phenotypic variance	0.48 (0.43, 0.54)	0.05 (0.01, 0.09)	0.21 (0.18, 0.25)	0.26 (0.21, 0.31)

Values are posterior means with the 95% highest posterior density interval shown in parentheses

Several animals showed a relatively high causal effect of the initial weight at the time of slaughter because of their (probably unintended) early slaughter. For example, 14 animals had coefficients of causal effect that were higher than 0.316, which indicates that about 10% of the phenotypic variance at slaughter was explained by the variance of initial weight. Consequently, since approximately 21% of the variance of initial weight could be explained by the reproduction farm effect (Table 3), about 2.1% of the variance of slaughter weight of these 14 animals could be explained by the effect of reproduction farm, which is not considered in the current model for genetic evaluation. Consequently, since these animals may affect the results, they should be eliminated from the genetic evaluation.

The estimated curve suggests that if slaughter of calves is planned at approximately 620 d after purchase, their growth can be recovered on the fattening farm if it was less than optimal on the reproduction farm. However, if animals are slaughtered earlier than 620 d, e.g., to reduce feeding costs, the causal effect of the initial weight will linearly increase as the slaughter age decreases (Fig. 2). For example, if the animal is slaughtered 440 d after purchase (approximately half a year earlier than 620 d), the effect of the initial phenotype increases to 0.405, suggesting that 16.4% of the phenotypic variance at slaughter is explained by the variance of initial weight. Thus, growth on reproduction farms should be considered with caution when selecting calves at market.

This is the first report on the causal effect of an initial phenotype on subsequent growth. However, rough estimates of this effect have been reported using multivariate analyses. For example, Meyer et al. [25] used four-variate analyses consisting of the birth, weaning (120–300 d after birth), yearling (301–500 d), and final (501–700 d) weights to estimate maternal genetic correlations in Hereford cattle and in a synthetic breed, and found that the correlations between weaning and yearling weights were 0.97 and 0.99 in Hereford and the synthetic breed, respectively, while those between weaning and final weights were 0.92 and 0.88, respectively. Similarly, Eler et al. [26] found that the maternal genetic correlation between weaning and yearling weights was 0.84 in Nelore cattle. In these studies, the maternal effects during the post-weaning periods were considered as a carry-over of those on the weaning weight. However, if the weaning weight is regarded as the initial phenotype, these correlations can be regarded as estimates of the causal effect. The causal effect that was estimated by the SGC model (Fig. 2) was lower than these estimates, which is likely due to differences in breeds and management conditions such as the type of feed.

### Genetic parameters

Estimates of the heritability and genetic correlations of the growth curve parameters ($A$, $B$, and $K$) are in Table 4. High heritability estimates were obtained for each parameter in all three models. By contrast, Takeda et al. [27] obtained much lower estimates i.e., 0.61, 0.08, and 0.17 for $A$, $B$, and $K$, respectively, in a population of Japanese black cattle. Two factors may explain this discrepancy: (1) the inference approach that was used, i.e., Takeda et al. [27] estimated the growth curve parameters and variance components for the parameters separately (two-step approach), whereas we estimated these simultaneously in our models; the two-step approach was previously shown to underestimate heritability because uncertainty in the growth curve parameter estimation is added to the residual variance in the subsequent variance component estimation for the growth curve parameters [28]; and (2) the number of records per animal, i.e., in [27], eight weight records per animal were available, whereas an average of 4.4 records were available for each animal in our study. A smaller number of records per animal may lead to an overestimation of the heritability because the growth curve parameters would tend to approach the prior means [i.e., ${\mathbf{x}}_{{\mathbf{i}}} {\varvec{\upbeta}}_{{\mathbf{A}}} + u_{i,A}$, ${\mathbf{x}}_{{\mathbf{i}}} {\varvec{\upbeta}}_{{\mathbf{B}}} + u_{i,B}$, and ${\mathbf{x}}_{{\mathbf{i}}} {\varvec{\upbeta}}_{{\mathbf{K}}} + u_{i,K}$ in Eqs. (4), (5), and (6)], resulting in decreased residuals ($e_{i,A}$, $e_{i,B}$, and $e_{i,K}$). To investigate the possibility that we overestimated the heritability, we conducted parametric bootstrapping, whereby the weights at slaughter were simulated with the parameter values estimated by the SGC model and the heritability was estimated using an animal model. We found that the mean heritability was 0.89 (± 0.09), compared to 0.73 for the heritability estimated from real records, which suggests that overestimation was an issue. Nevertheless, since the observed heritability estimate was higher than the lower 5% quantile of the simulated values (0.71), we consider that the heritability estimates generated in our study did not deviate substantially from the true values. Because heritabilities of body weight of Japanese Black cattle are often relatively high (e.g., 0.61 [29] and 0.56 [30] for carcass weight), high heritabilities for growth curve parameters would be plausible. However, we also admit that the heritabilities estimated in this study are higher than expected, and thus, further investigations are required to verify this issue.

**Table 4:** Heritability (diagonal), genetic correlation (lower triangular), and residual correlation (upper triangular) of the growth curve parameters *A*, *B*, and *K* estimated by each model

		$A$	$B$	$K$
SGC^a^	$A$	0.97 (0.90, 1.00)	0.34 (− 0.20, 0.83)	− 0.57 (− 0.96, − 0.05)
	$B$	0.63 (0.57, 0.69)	0.91 (0.84, 0.97)	− 0.30 (− 0.79, 0.22)
	$K$	− 0.69 (− 0.73, − 0.64)	− 0.67 (− 0.76, − 0.58)	0.91 (0.78, 0.99)
GC_A^b^	$A$	0.96 (0.89, 0.99)	0.35 (− 0.25, 0.83)	− 0.42 (− 0.92, 0.20)
	$B$	0.69 (0.59, 0.77)	0.66 (0.53, 0.78)	− 0.26 (− 0.76, 0.34)
	$K$	− 0.77 (− 0.81, − 0.73)	− 0.67 (− 0.75, − 0.58)	0.96 (0.91, 0.99)
GC_B^c^	$A$	0.94 (0.89, 0.99)	0.61 (0.21, 0.91)	− 0.51 (− 0.93, 0.03)
	$B$	− 0.30 (− 0.44, − 0.17)	0.69 (0.55, 0.84)	− 0.28 (− 0.78, 0.30)
	$K$	− 0.75 (− 0.79, − 0.71)	0.65 (0.55, 0.74)	0.95 (0.91, 0.99)

Values are posterior means, with the 95% highest posterior density interval shown in parentheses

^a^Structural growth curve model fitted to the data with the entry day to the experimental station set as the initial day

^b^Growth curve model fitted to the data with the entry day to the experimental station set as the initial day

^c^Growth curve model fitted to the data with the birth day set as the initial day

The genetic correlations between growth curve parameters differed according to the model that was used. The correlations between $A$ and $B$ were positive in the SGC and GC_A models but negative in the GC_B model, whereas the correlations between $B$ and $K$ were negative in the SGC and GC_A models but positive in the GC_B model. These opposite tendencies may be due to the day of entry in the stations being set as the initial day in the SGC and GC_A models, although the actual age of the calves’ at entry differed (251.5 ± 20.4 d). Because parameter $B$ shifts the growth curve back and forth, setting the entry day as the initial day would affect the estimates of $B$ and its correlation with the other parameters. Similar contrasting results in genetic correlations were also found in two independent pig studies: Koivula et al. [8] reported strong negative genetic correlations between $A$ and $K$ (− 0.80) and between $B$ and $K$ (−0.80) but a positive correlation between $A$ and $B$ (0.88), whereas Coyne et al. [9] reported negative correlations between $A$ and $B$ (− 0.69) and between $A$ and $K$ (− 0.78) but a positive correlation between $B$ and $K$ (0.76). Although the estimates in the latter study [9] differed depending on the method used for estimation, a negative correlation between $A$ and $B$ was consistently observed. Interestingly, Koivula et al. [8] used test age, starting from when the body weight was approximately 30 kg, which may have affected the estimation of the genetic correlation, as observed in our study. It will be difficult to determine which day (birth or entry) is most valid as the initial day, and this may depend on the data. However, these findings indicate that genetic correlations between the parameters must be interpreted with caution.

The interpretation of genetic correlations is also complicated by compensation between the parameters. For example, $K$ which increases the maximum growth speed, also increases the mature weight controlled by $A$. Therefore, when fitting a curve to a measured value of mature weight, $K$ should decrease as $A$ increases, and vice versa, resulting in a negative correlation between the two. This may explain why a negative correlation was consistently observed between these parameters in previous studies [8, 9] and the present study. Thus, the biological interpretation of the genetic correlation between growth curve parameters may be controversial.

## Conclusions

By fitting our newly developed SGC model to data on weight of beef cattle, we were able to estimate the causal effect of the initial weight (weight on the day of entry to the stations) on growth. Because all of the evaluated criteria supported the proposed model, we suggest that the SGC model can provide more accurate estimates of the genetic effects on growth, particularly for the cattle cohort that was assessed in this study. Moreover, our data suggest that the inferred curve of the causal effect can provide valuable information for planning the time of slaughter, building models for genetic evaluation, and selecting calves at markets.

## Additional files

**Additional file 1.** Phenotype (weight) records of 3831 animals after birth. The first row is days after birth (i.e., age). Missing values are 999,999.

**Additional file 2.** Phenotype (weight) records of 3831 animals after entry to experimental stations. The first row is days after entry to experimental stations. Missing values are 999,999. The order of animals is the same as in Additional file 1.

**Additional file 3.** Design matrix for fixed effects on weight at entry. The first row is name of effects. The order of animals is the same as in in Additional file 1.

**Additional file 4.** Design matrix for fixed effects on growth curve parameters. The first row is name of effects. The order of animals is the same as in Additional file 1.

**Additional file 5.** Inverse of ${\mathbf{A}}$ matrix. Only non-zero elements are presented as [RowNumber ColumnNumber Value].

**Additional file 6.** Reproduction farm numbers. The order of animals is same as in in Additional file 1.

**Additional file 7.** Correspondence of row numbers between Additional file 1 and Additional file 5 for animal effects (i.e., breeding values).

**Additional file 8.** Correspondence of row numbers between Additional file 1 and Additional file 5 for maternal effects.
